# Lambs immunized with an inactivated variant of *Anaplasma phagocytophilum*

**DOI:** 10.1186/s13028-015-0131-1

**Published:** 2015-07-25

**Authors:** Snorre Stuen, Wenche Okstad, Karin Artursson, Basima Al-Khedery, Anthony Barbet, Erik G Granquist

**Affiliations:** Department of Production Animal Clinical Sciences, Norwegian University of Life Sciences, Kyrkjevegen 332/334, 4325 Sandnes, Norway; Department of Bacteriology, National Veterinary Institute, SE-751 89 Uppsala, Sweden; Department of Infectious Diseases and Pathology, University of Florida, Gainesville, FL 32611 USA; Department of Production Animal Clinical Sciences, Norwegian University of Life Sciences, PO Box 8144, 0033 Oslo, Norway

**Keywords:** *Anaplasma phagocytophilum*, Immunization, Sheep

## Abstract

**Background:**

*Anaplasma phagocytophilum* (formerly *Ehrlichia phagocytophila*) is an obligate intracellular bacterium causing the disease tick-borne fever (TBF) in domestic ruminants. An effective vaccine against the infection has been demanded for livestock by sheep farmers and veterinary practitioners for years.

**Findings:**

In the present study, we immunized lambs with an inactivated suspension of 1 × 10^8^ killed *A. phagocytophilum* organisms mixed with adjuvant (Montanide ISA 61VG; Seppic). Twelve 9-months-old lambs of the Norwegian White Sheep breed were used. A full two-dose series of immunization was given subcutaneously to six lambs with a 4 week interval between injections. One month after the last immunization, all lambs were challenged with the homologous viable variant of *A. phagocytophilum*. After challenge, all lambs showed clinical responses for several days, although the immunized lambs reacted with an anamnestic response, i.e. significant reduction in infection rate and a significantly higher antibody titer.

**Conclusion:**

Immunization with inactivated *A. phagocytophilum* did not protect lambs TBF.

## Findings

Tick-borne fever (TBF) caused by the bacterium *Anaplasma phagocytophilum* (formerly *Ehrlichia phagocytophila*) is an endemic disease of sheep in tick (*Ixodes ricinus*) infested areas of Norway [1]. TBF has for decades been one of the main scourges for the sheep industry in the coastal areas of Norway. An effective vaccine against the infection has been demanded by sheep farmers and veterinary practitioners in Norway for years. However, there are currently no vaccines available against TBF. In endemic areas, prophylactic use of long-acting tetracycline, regular dipping or pour-on treatment with pyrethroids are used extensively [2]. However, there is a growing concern about the environmental safety and human health, increasing resistance in bacteria and their vectors related to antibiotics and chemical controls of ticks [3].

In the present study we investigated if an inactivated crude antigen based on inactivated bacteria from buffy coat extracts could protect lambs upon challenge with live *A. phagocytophilum*.

Twelve unexposed 9-months-old lambs of the Norwegian White Sheep breed were used. All lambs belonged to the experimental sheep flock at the Department of Production Animal Clinical Sciences and were housed indoors during the trial. Two groups of lambs with mixed gender and equal distribution of mean live weight were established. The experimental study was ethically approved by the National Animal Research Authority (Norway).

The strain of *A. phagocytophilum* used originated from an infected lamb in a Norwegian sheep flock known to have problems with TBF. Based on partial sequencing of the *16S rRNA* gene, the variant of *A. phagocytophilum* was identical to GenBank accession number M73220. This variant has previously been evaluated in several infection studies [4, 5], and infected heparinised blood was stored at −70°C with 10% dimethyl sulphoxide (DMSO). The batch of inoculum was used for antigen preparation and in the later infection challenges.

In order to obtain a sufficient amount of bacterial inoculum, one unexposed lamb was infected intravenously with 2 ml of a heparinized DMSO-stabilate of *A. phagocytophilum*. On the second day of fever (day 5 after inoculation), 300 ml Na-citrated blood was collected from this lamb and the buffy coat was obtained at 4–6°C, by centrifugation in a swing-bout bucket rotor (Heraeus Multifuge 3S-R, Termo Sci. Germany) at 2,500×*g* for 30 min. The isolated buffy coat was washed three times in 1× PBS at 1,500×*g* for 20 min, and re-suspended in PBS after the last centrifugation. Quantification of the bacterial content in the buffy coat was determined by qPCR [6]. The buffy coat was frozen in 10 ml aliquots at −70°C for further analysis.

For antigen preparation, 10 ml frozen buffy coat containing approximately 8 × 10^8^ copies of *A. phagocytophilum* per ml was used. The material was inactivated using 0.3% formaldehyde [7] for 48 h at room temperature. Thereafter, the material was tested for lack of infectivity by intravenous inoculation into two naive lambs (data not shown).

The final preparation was made by mixing 1 ml inactivated buffy coat and 1 ml adjuvant (Montanide ISA 61 VG, Seppic). The antigen solution and the mineral oil adjuvant were mixed to water in oil emulsion using two syringes connected by a three way valve [7]. The final antigen dose contained approximately 1 × 10^8^ inactivated *A. phagocytophilum* and was used immediately after preparation.

Six lambs were immunized subcutaneously twice (one month apart) with the inactivated crude antigens. One month after the last immunization, all lambs were infected intravenously with 2 ml of the homologous viable batch of *A. phagocytophilum* with an approximate infection dose equal to 0.5 × 10^6^ infected neutrophils per ml. A similar dose has earlier been used in other *A. phagocytophilum* infection studies [1, 4].

The lambs were clinically observed daily and the rectal temperature was measured, starting on the first day of immunization [5]. Blood samples (EDTA) were collected every third day for the first 14 days after each immunization and then daily during the fever period following the inoculation of infective blood. After the fever had subsided, blood samples were collected on a weekly basis. From these EDTA-blood samples haematological values including total and differential leucocyte counts were determined electronically (Technicon H1^®^, Miles Inc., USA) and blood smears were prepared and stained with May-Grünwald Giemsa [5].

In order to detect *A. phagocytophilum* infection EDTA-blood samples were also analysed for *Anaplasma*-DNA by qPCR [6]. In addition, sera were collected every second week post each immunization and after challenge on days 0, 7, 14, 21, 28, 42, and 63. Sera were analyzed using an indirect immunofluorescence antibody assay (IFA) to determine the antibody titers to an equine variant of *A. phagocytophilum* (formerly *Ehrlichia equi*) [8].

Statistical calculations were done using Statistix, version 4.0 (Analytical Software), and a two-sample *t* test was used to compare clinical, haematological and serological variables. A *P* value of <0.05 was considered significant.

No clinical signs or haematological changes were observed after immunization. However, all immunized lambs reacted with a firm palpable subcutaneous nodule without abscess formation at the site of inoculation, starting 3–4 days after each immunization which disappeared about 4 weeks post immunization.

**Fig. 1:**
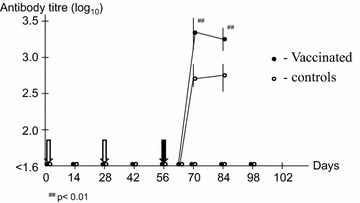
Antibody titers in *A. phagocytophilum*-immunized (days 0 and 28) (*white arrow*) and control lambs. All lambs were challenged with live bacteria on day 56 (*black arrow*). A titer below 1.6 (<1/40) was considered negative.

**Fig. 2:**
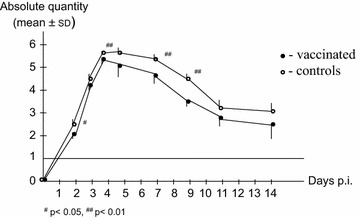
*A. phagocytophilum* infection in vaccinated and control lambs post infection (quantitative PCR). The *horizontal line* is the threshold of bacteraemia (10 copies). The results are presented as logarithm transformed Cq readings (X), calculated as log10 (1 + X).

After challenge, all lambs reacted with fever, bacteraemia, neutropenia and an antibody response typical of an *A. phagocytophilum* infection [4]. Although the result indicates a difference in the clinical and haematological variables, no significant differences were obtained (data not shown). However, there was a significant difference (*P* < 0.01) in level of bacteraemia (from days 4–9) and the antibody responses between immunized and control groups (Figs. 1, 2). After challenge, relapses of fever for 1–3 days occurred in two (33%) and five (83%) of the immunized and control lambs, respectively.

In the present study, no serologic response was observed after immunization. Lack of seroconversion observed in the immunized lambs could be due to low immunogenicity to the antigens used. However, the present serological test has earlier been used successfully when lambs were infected with the currently described variant of *A. phagocytophilum* [4]. Lack of detectable immune response could also be due to a low dose of antigen, masking of epitopes by formaldehyde treatment or the adjuvant used. Montanide ISA and formaldehyde have earlier been included in vaccine preparations [7, 9], and a similar dose of antigen was used in a vaccination study with the related organism *Ehrlichia ruminantium* [10].

After challenge, there were no significant differences in temperature reaction or the differential leucocyte counts between the two groups of lambs, although significant differences (*P* < 0.01) were observed in infection levels and antibody responses. The increased number of fever relapses in the unimmunized lambs, indicates a more solid immunity to *A. phagocytophilum* after immunization [11]. These results indicate an anamnestic response, although too small to give protective immunity.

Immunity after experimental infection with a live variant of *A. phagocytophilum* varies from weeks to years [1]. *A*. *phagocytophilum* are obligate intracellular pathogens and cellular immunity is in general necessary for an effective immunity against such organisms [11]. However, antibodies to rickettsial infections have been shown to block the initial adhesion and penetration of the bacterium, enhance phagosome-lysosome fusion and phagocytosis followed by destruction of the organisms [12, 13].

An earlier observation indicates that specific antibodies could induce protection from *Anaplasma* infection. In one trial, mice were either vaccinated with a lysate of human variant of *A. phagocytophilum* (HGA-agent) or were given HGA-antisera directly from vaccinated mice. After challenge with the same variant, these mice were partially protected, indicating that antibodies are sufficient to protect substantially, but not fully against infection [14].

Crude preparation of the bacteria may expose mainly dominant antigens giving poor protection against disease due to irrelevant antigens derived either from the agent itself or from material used to produce it [15], although no detectable serological response was obtained after a similar immunization trial in lambs, using a purified cell-cultured variant of *A. phagocytophilum* (Stuen, unpublished results).

In order to develop a successful vaccine, the challenge is to choose shared or subdominant antigens that are conserved amongst all strains of *A. phagocytophilum* and to produce these in sufficient quantity [16–18]. Genome sequencing of multiple strains [19] may be required to identify conserved antigens. Further research to develop sub-unitvaccines or live vaccine candidates should therefore focus on promoting the expression of sub-dominant surface proteins of *A. phagocytophilum,* as described in recent studies on the related organism *A. marginale* [20–22].

In conclusion, immunization with antigens based on the whole bacterium did not protect lambs from an *A. phagocytophilum* infection. After challenge, all lambs showed clinical responses for several days, although the immunized lambs had reduced levels of infection. Improved antigens are necessary in order to obtain protection from bacteraemia and clinical manifestation of tick-borne fever.
